# Molecular Epidemiology of *Staphylococcus epidermidis* Implicated in Catheter-Related Bloodstream Infections at an Academic Hospital in Pretoria, South Africa

**DOI:** 10.3389/fmicb.2018.00417

**Published:** 2018-03-07

**Authors:** Marthie M. Ehlers, Wilhelmina Strasheim, Michelle Lowe, Veronica Ueckermann, Marleen M. Kock

**Affiliations:** ^1^Department of Medical Microbiology, Faculty of Health Sciences, University of Pretoria, Pretoria, South Africa; ^2^National Health Laboratory Service, Tshwane Academic Division, Pretoria, South Africa; ^3^Department of Internal Medicine, University of Pretoria, Pretoria, South Africa

**Keywords:** *Staphylococcus epidermidis*, catheter-related bloodstream infections, South Africa, SCC*mec* typing, PFGE, MLST, ST596, ST2

## Abstract

*Staphylococcus epidermidis* is one of the most prevalent pathogens implicated in catheter-related bloodstream infections (CRBSI) at an academic hospital in Pretoria, South Africa. Little is known about the clonality and the prevalence of antibiotic resistance and virulence genes in *S. epidermidis* (e.g., *ica*AB, IS*256, mec*A, and *qac*A/B). A total of 508 intravascular catheters (IVCs) from 331 patients were submitted for culture from May to October 2013. Only 50% (*n* = 253/508) of the IVCs were accompanied by blood cultures (BCs) taken within 48 h. Forty-four percent (44%; *n* = 112/253) of IVCs were colonised, of which 26% (*n* = 65/253) were associated with a CRBSI. We identified *S. epidermidis* as the causal agent in 31% (*n* = 20/65) of the CRBSI cases. Fifty-nine *S. epidermidis* isolates were obtained, 23 isolates were cultured from 22 IVCs and 36 isolates were cultured from 36 BCs. All *S. epidermidis* isolates were resistant to β-lactams (100%; *n* = 59/59), followed by high levels of resistance toward erythromycin (86%; *n* = 51/59) and gentamicin (81%; *n* = 49/59). The *mec*A gene was prevalent in all the (100%, *n* = 59/59) isolates. Isolates contained the IS*256* element (83%, *n* = 49/59), the *ica*AB gene (81%, *n* = 48/59) and, the *qac*A/B gene (81%, *n* = 48/59). All 48 isolates were *qac*A positive upon restriction enzyme digestion of the *qac*A/B amplicons. Phenotypic resistance toward 0.5% (m/v) chlorhexidine was not observed. Staphylococcal Cassette Chromosome (SCC) *mec* typing showed that SCC*mec* type IV (31%; *n* = 18/59) was the most prevalent. The remaining SCC*mec* elements were highly diverse. Pulsed-field gel electrophoresis (PFGE) showed that *S. epidermidis* isolates from individual patients were mostly clonal. Multilocus sequencing typing (MLST) of 10 sequenced isolates showed that sequence type (ST) 2 (40%; *n* = 4/10) was the most frequently detected, followed by ST54 (20%; *n* = 2/10), ST28 (10%; *n* = 1/10), ST59 (10%; *n* = 1/10) and ST490 (10%; 1/10). One isolate was newly assigned to ST596. These *S. epidermidis* infections can be attributed to patients' skin microflora or to poor infection control practices. Currently, *S. epidermidis* strains circulating in the studied hospital are multidrug-resistant and highly adaptive to environmental changes.

## Introduction

Catheter-related bloodstream infections are defined by the Infectious Diseases Society of America (IDSA) as bacteraemia or fungaemia in a catheterised febrile patient, with at least a positive blood culture (BC) and intravascular catheter (IVC) culture, yielding the same microorganism, with no other possible source of infection besides the IVC (Mermel et al., 2009). If the isolated microorganism is a common commensal, at least two positive blood cultures are required to exclude the possibility of contamination (Mermel et al., 2009). *Staphylococcus epidermidis* is a classic example of a common commensal and is an ever-present coloniser of the human skin (Percival et al., 2012). However, *S. epidermidis* can become an opportunistic pathogen if the external barrier of the skin surface is breached, such as during the insertion of an IVC (Schoenfelder et al., 2010).

Biofilm formation, encoded by the *ica*ADBC operon, is central to the pathogenesis of *S. epidermidis* (Otto, 2009). The biofilm allows the bacterium to colonise the IVC and subsequently seed into the bloodstream (Otto, 2008). Certain *S. epidermidis* strains may have an evolutionary advantage due to methicillin-resistance facilitated by the *mec*A gene, phenotypic variation caused by insertion sequence IS*256* and genotypic resistance to antiseptics, such as chlorhexidine, mediated by efflux pumps encoded by the *qac*A/B genes (Otto, 2009; Schoenfelder et al., 2010; Horner et al., 2012). As a result, certain strains may be resistant to infection prevention practices, able to evade host defences and subsequently cause disease (Otto, 2009).

Previously, *S. epidermidis* was identified as the most common CRBSI pathogen at an academic hospital in Pretoria, South Africa (Strasheim et al., 2015). This study molecularly characterised and genotyped *S. epidermidis* isolates implicated in CRBSI events and described *S. epidermidis* isolates from South Africa for the first time.

## Materials and methods

### Study setting and isolate collection

The IVCs and BCs were submitted from an 832-bed academic hospital in Pretoria, South Africa, over a six-month period (May to October 2013) to the diagnostic division of the Department of Medical Microbiology [Tshwane Academic Division, National Health Laboratory Service (NHLS)]. The laboratory processes specimens from academic hospitals as well as district hospitals and various clinics as part of standard care. Routine analysis was performed as requested by a medical practitioner on the laboratory requisition form and according to sample type. A *S. epidermidis* CRBSI was defined as the simultaneous isolation of the bacterium from an IVC and one or more BCs, which were submitted for culture within 48 h of each other (Mermel et al., 2009; Conrick-Martin et al., 2013). Even though a single positive BC yielding *S. epidermidis* was likely to present contamination, we included these samples due to the lack of local BC practices. In practice, compliance to guidelines were suboptimal in the studied hospital, since catheters were rarely accompanied by BCs. If a BC accompanied a catheter, often only one BC was submitted. Patient demographics [i.e., age, sex, ward, underlying conditions, length of hospitalisation, antimicrobial exposure and the presence of specific risk factors (HIV-status, diabetes, chronic renal failure, malnutrition, loss of skin integrity, neutropenia and total parenteral nutrition administration) for CRBSI] and catheter details (i.e., length of catheterisation, catheter type, vessel occupied, insertion site, pathway followed from insertion site to vessel and the number of lumens) were collected.

### *Staphylococcus epidermidis* isolation, identification, and susceptibility testing

Intravascular catheter tips were cultured according to the semi-quantitative roll-plate method (Maki et al., 1977). Blood cultures were processed with the BacT/ALERT 3D system (bioMérieux, France). Identification and antimicrobial susceptibility testing of *S. epidermidis* isolates were performed with the VITEK® 2 automated system (bioMérieux, France). The minimum inhibitory concentration (MIC) was recorded according to the 2013 Clinical Laboratory Standards Institute (CLSI) guidelines (Clinical Laboratory Standards Institute, 2013). Identification of *S. epidermidis* isolates was confirmed by matrix-assisted laser desorption/ionisation time of flight mass spectrometry (MALDI-TOF MS) (Bruker Daltonics, USA) along with a conventional identification (ID) polymerase chain reaction (PCR) amplification.

### Total DNA extraction

Total DNA of the *S. epidermidis* isolates was extracted with the ZR Fungal/Bacterial DNA MiniPrep™ kit (Zymo Research, USA), with some modifications to the manufacturer's instructions. A volume of 2 mL of overnight Brain Heart Infusion broth (Merck, Germany) was used as starting material for the extractions. Beta-mercaptoethanol [0.5% (*v/v*)] (Merck, Germany) was added to the DNA binding buffer (Zymo Research, USA). A volume of 600 μL of Lysis solution (Zymo Research, USA), instead of the recommended 750 μL of Lysis solution (Zymo Research, USA), was used in the ZR BashingBead™ Lysis Tubes (Zymo Research, USA). The DNA obtained was used in all downstream PCR applications.

### Multiplex PCR assay to identify *Staphylococcus epidermidis*

This first multiplex-PCR (M-PCR) assay detected five staphylococcal species simultaneously. The genes targeted in the M-PCR assay included an internal fragment of *S. epidermidis* [124 base pair (bp)] (Martineau et al., 1996), the nuclease (*nuc*) gene of *S. aureus* [359 bp], *S. capitis* [525 bp], and *S. hominis* [177 bp] (Hirotaki et al., 2011), and the mevalonate pathway (*mva*A) gene of *S. haemolyticus* [271 bp] (Pereira et al., 2010). The 16S ribosomal (rRNA) gene for the *Staphylococcus* genus [597 bp] was included as an internal control (Al-Talib et al., 2009). A final concentration of 0.2 μM was used for each primer in the reaction mixture. The M-PCR reaction mixture consisted of 12.5 μL of 2 × QIAGEN® Multiplex PCR Master Mix (QIAGEN®, Netherlands), 2.5 μL of the 10 × primer mixture, 2.0 μL of template DNA and 8 μL of nuclease-free water. Amplification was done in a G-STORM™ thermocycler (Somerton Biotechnology Centre, UK). The cycling conditions included an initial *Taq* polymerase activation step (95°C for 15 min), followed by 30 cycles of denaturation (94°C for 30 s); annealing (58°C for 90 s) and extension (72°C for 90 s) and a final extension step (68°C for 15 min). The PCR amplicons were visualised on a 1.8% (m/v) MetaPhor™ agarose (Lonza, USA) gel, stained with 5 μL of ethidium bromide (10 μg/mL) (Sigma-Aldrich, USA). The amplicons were visualised under ultraviolet (UV) light (Transilluminator, Ultraviolet Products Incorporated, USA) and all visible bands were manually compared to a 50 bp DNA ladder (ThermoScientific, USA).

### Multiplex PCR assay for the detection of is*256, ica*A/B, *mec*A, and *qac*A/B genes

*S. epidermidis* isolates were tested for the presence of the IS*256* [762 bp] (Koskela et al., 2009), *ica*AB [546 bp] (Iorio et al., 2011), *mec*A [310 bp] (McClure et al., 2006) and *qac*A/B [220 bp] (Sekiguchi et al., 2004) genes, using a second M-PCR assay. Primer concentrations for the IS*256, ica*AB, *mec*A and *qac*A/B genes were 0.2, 2.0, 1.0, and 0.2 μM in the 10 × primer mix, respectively, which was prepared according to the recommendations of the manufacturer (QIAGEN®, USA). Each 25 μL reaction mixture contained 12.5 μL of QIAGEN® Multiplex PCR Master Mix, 2.5 μL of the 10 × primer mix, 2.5 μL of template DNA and 7.5 μL of nuclease-free water. Amplification was done in a G-STORM™ thermocycler (Somerton Biotechnology Centre, UK). The cycling conditions included an initial *Taq* polymerase activation step (95°C for 15 min), followed by 35 cycles of denaturation (94°C for 30 s); annealing (57°C for 90 s) and extension (72°C for 90 s) and a final extension step (72°C for 10 min). The detection and visualisation steps were repeated as described in the previous section, except for this assay a 2% (m/v) SeaKem® LE agarose (Lonza, USA) gel was used. A 50 bp DNA ladder (ThermoScientific, USA) was used as a marker. One positive amplicon for each of the screened genes was sequenced in both forward and reverse directions by Inqaba Biotechnical Industries, Pretoria, South Africa. These confirmed positive genes were used as positive controls in the M-PCR assays. A negative control [nuclease-free water (Qiagen, Germany)] was included for all M-PCR assays.

### Enzyme digestion to distinguish between the *qac*A and-B gene

The *qac*A/B primers used in the M-PCR assays did not distinguish between the *qac*A and -B genes, since these genes are highly homologous and differ only at seven nucleotides (Sekiguchi et al., 2004; Horner et al., 2012). The method published by Sekiguchi et al. (2004) was followed to distinguish between the *qac*A [220 bp] and -B [44 bp and 176 bp] genes. The following modifications were made: (i) Instead of using the prescribed 5 U of *Alu*I, 0.25 μl of the *Alu*I restriction enzyme (New England Biolabs, USA) and 1 μl of the cut-smart buffer (New England Biolabs, USA) were added to 5 μl of the amplified products (a singleplex PCR assay was performed to detect the *qac*A/B genes. The cycling conditions was the same as described in the previous section); (ii) a 3.5% (m/v) MetaPhor™ agarose gel (Lonza, USA) was prepared instead of the suggested 15–25% polyacrylamide gel. The amplified products were digested at 36 ± 1°C for 90 min. The bands were visualised and captured as described elsewhere. A 50 bp DNA ladder (ThermoScientific, USA) was used as a marker. One positive amplicon form each of the screened genes were sequenced in both forward and reverse directions by Inqaba Biotechnical Industries, Pretoria, South Africa. The confirmed positive gene was used as a positive control. A negative control [nuclease-free water (Qiagen, Germany)] was included for all M-PCR assays. The identity of the genes was confirmed using BLAST after sequencing of the products.

### Staphylococcal cassette chromosome *mec* typing

The M-PCR assays for the *ccr* and the *mec* gene complexes was performed as described by Kondo et al. (2007). Three additional primers (ccrCU1; α4U and β4U) were added to the *ccr* gene complex M-PCR assay to detect all known *ccr*C allotypes, as well as the *ccr*AB4 gene complex (Ruppé et al., 2009). The *ccr* and the *mec* gene complexes were classified as recommended by the guidelines from the International Working Group on the Classification of Staphylococcal Cassette Chromosome (SCC) elements (IWG-SCC) (Ito et al., 2009). SCC *mec* types were assigned based on the Roman numeral system, however the latter was not assigned to novel *ccr* and *mec* gene complex combinations if it was discovered in other methicillin-resistant staphylococci. These SCC*mec* types were reported using the raw combination of the *ccr* and *mec* gene complexes.

### Pulsed-field gel electrophoresis

Plug preparation of *S. epidermidis* isolates were done according to the unified PFGE protocol for Gram-positive bacteria developed by the Division of Health Care Quality Promotion from the Centers for Disease Control and Prevention (CDC) (http://www.cdc.gov/hai/pdfs/labSettings/Unified_PFGE_Protocol.pdf) with some modifications. The modifications included an overnight lysis step of the plugs at 51°C, instead of the 2-h lysis step at 54°C as described by the protocol. The *Sma*I restriction digested plugs were separated by PFGE using the Rotaphor® system 6.0 (Biometra, Germany) in a 1.2% (m/v) SeaKem LE (Lonza, Rockland, USA) agarose gel in 0.25 × TBE (Sigma-Aldrich, USA) buffer. The programmed parameters for electrophoresis were set at 220 V changing linearly to 200 V at a constant angle of 120°. The switch time was set at 5 s linearly to 40 s (McDougal et al., 2003). The gel ran for 25 h at 13°C. The gel was stained with ethidium bromide (0.25 μg/mL) (Sigma-Aldrich, USA) for 15 min and destained in ultrapure water for 30 min. The destained gel was visualised under UV light (Transilluminator, Ultra-violet Products Incorporated, USA) and photographed with the UVP GelDoc-It system (Transilluminator, Ultra-violet Products Incorporated, USA). Pulsed-field gel electrophoresis patterns were analysed with the GelCompar *II* system (Applied Maths, Belgium) and the percentage of relatedness was determined by the Dice Coefficient. Isolates from IVC tips and BCs were considered genetically-related if the pulsotype's banding patterns showed ≥ 80% similarity (Applied Maths, Belgium), which corresponds to the Tenover criteria (possibly related with 4–6 band differences) (Tenover et al., 1995; Peirano et al., 2014). Representatives from each major PFGE pulsotype (≥ 5 isolates), minor pulsotypes (< 5 isolates) with ≥ 80% similarity and selected singletons were chosen for MLST analyses.

### Multilocus sequence typing

Multilocus sequence typing was performed as described by Thomas et al. (2007). Ten representative *S. epidermidis* isolates were selected from three major pulsotypes (*n* = 4), three minor pulsotypes (*n* = 3) and three singletons (*n* = 3). Sequencing of the seven housekeeping genes (*arc*C, *aro*E, *gtr, mut*S, *pyr*R, *tpi*, and *yqi*L) was done by Inqaba Biotechnical Industries (Pretoria, South Africa). Originally, the *S. epidermidis* MLST database (http://sepidermidis.mlst.net/) was used, [hosted now by pubMLST (https://pubmlst.org/sepidermidis/)] provided allelic profiles and the subsequent STs. A comparative electronic Based Upon Related Sequence Types (eBURST) v3 analysis (http://eburst.mlst.net/) was run to infer a hypothetical pattern of evolutionary descent. All STs were submitted to the curator (Jonathon Thomas, email address: miragaia@itqb.unl.pt) of the *S. epidermidis* database.

## Results

### Prevalence of *Staphylococcus epidermidis* catheter-related bloodstream infection cases

A total of 508 IVCs from 331 patients were submitted for culture during the study period. Only 50% (*n* = 253/508) of the IVCs were accompanied by BCs taken within 48 h. Forty-four percent (44%; *n* = 112/253) of these IVCs accompanied by a BC were colonised, of which 25,6% (*n* = 65/253) of the colonised IVCs were associated with a CRBSI. *S. epidermidis* was implicated as the aetiological agent in 31% (*n* = 20/65) of the CRBSI cases of which 59 *S. epidermidis* were obtained, 23 isolates were cultured from 22 IVCs and 36 isolates were cultured from 36 BCs. Nine patients (four adults and five paediatric patients) had a single positive BCs for *S. epidermidis*. The pulsotypes obtained from the IVC and the BC for these 9 patients were compared.

### Clinical details of patients with a *Staphylococcus epidermidis* catheter-related bloodstream infection

Patients with an *S. epidermidis* CRBSI were mostly male (65%; *n* = 13/20). Adult patients (75%; 15/20) were more often affected than paediatric patients (25%; *n* = 5/20). Adult patients were on average 43 years [standard deviation (SD) = ± 14 years] old, whereas paediatric patients were on average 31 days (*SD* = ±16 days, outlier of 1 year) old. Sixty percent (60%; *n* = 12/20) of *S. epidermidis* CRBSI cases were from patients in an intensive care unit (ICU) and 40% (*n* = 8/20) were from patients located in other wards throughout the hospital. Thirty-five percent (40%; *n* = 8/20) of these patients had an underlying gastroenterological condition, followed by 30% (*n* = 6/20) of patients with either an underlying nephrological condition or a traumatic incident. The clinical condition of one patient was unknown. The remaining patients had a diverse range of underlying conditions [i.e., cardiovascular (5%; *n* = 1/20), dermatology (5%; *n* = 1/20), neurologic (5%; *n* = 1/20), surgery (5%; *n* = 1/20) and respiratory (5%; *n* = 1/20)].

On average, patients were exposed to two or more antimicrobials. Fifty-five percent (55%; *n* = 11/20) of patients with *S. epidermidis* CRBSI had meropenem exposure, followed by 30% (*n* = 6/20) for both vancomycin and colistin, and 15% (*n* = 3/20) to tazobactam. The presence of specific risk factors for the development of a CRBSI was unknown for a single patient (*n* = 19). Fifty-eight percent (58%; *n* = 11/19) of patients had previously received total parenteral nutrition and 16% (*n* = 3/19) of patients had HIV, diabetes or renal failure as a risk factor. Twenty-one percent (21%; *n* = 4/19) of patients were either malnourished or had lost the integrity of their skin. A single patient (5%; *n* = 1/19) had neutropaenia as a risk factor. Patients were hospitalised on average for 20 days (*SD* = ± 14 days), prior to the development of an *S. epidermidis* CRBSI.

### Characteristics of the intravascular catheters implicated in a *Staphylococcus epidermidis* catheter-related bloodstream infection

A total of 22 IVC catheters were involved in the *S. epidermidis* CRBSI events. All IVCs were short non-cuffed lines and were not impregnated with antimicrobials. Seventy-three percent (73%; *n* = 16/22) of the IVCs submitted were central venous pressure (CVP) tips, followed by 14% (*n* = 3/22), 9% (*n* = 2/22), and 5% (*n* = 1/22) of IVCs being VasCath lines, arterial catheters and Broviac lines, respectively.

The duration of catheterisation, the vessel occupied, the insertion site, the pathway followed from the insertion site to the vessel and the number of lumens was unknown for a single CVP and thus was excluded (*n* = 15). The CVPs were placed either in the subclavian vein (67%; *n* = 10/15) or the internal jugular vein (33%; *n* = 5/15). All CVPs placed in the subclavian vein were non-tunnelled. Two of the subclavian vein CVPs (20%; *n* = 2/10) had a double-lumen, whereas the rest (80%; *n* = 8/10) had a triple-lumen. All the CVPs placed in the internal jugular vein had a triple-lumen and the pathway followed under the skin was non-tunnelled. The remaining CVPs were in place for an average of 12 days (*SD* = ± 6 days).

All VasCath lines were placed centrally in the internal jugular vein. The two triple-lumen VasCath lines were non-tunnelled, whereas the double-lumen VasCath line was tunnelled. The duration of placement was unknown for a single VasCath line, whereas the other two VasCath lines were in place for an average of 7 days. Both arterial catheters were short, single-lumen, non-tunnelled and placed in a radial vein. One of the arterial catheters was simultaneously in place with a CVP. The one arterial catheter was in place for 10 days prior to the development of *S. epidermidis* CRBSI. The short, double-lumen, non-tunnelled Broviac line was in place for 14 days centrally in the subclavian vein.

### Identification, antimicrobial susceptibility profiles, distribution of the *icaAB, IS256, mecA, and qacA/B* genes, restriction enzyme digestion of the *qac*A/B genes and chlorhexidine susceptibility testing

**Figure 1:**
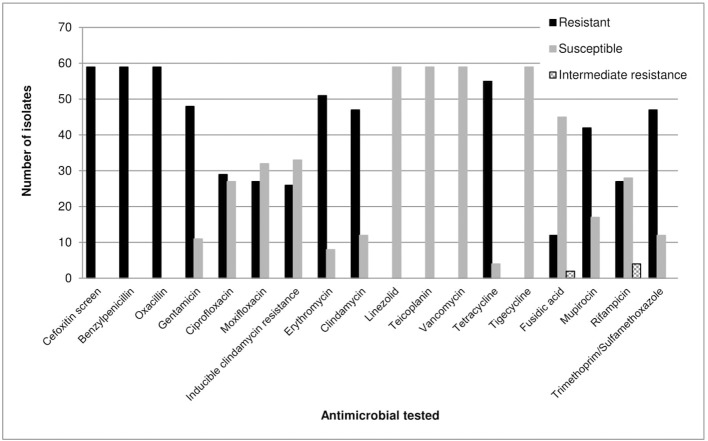
Antimicrobial susceptibility profiles of 59 *S. epidermidis* isolates as determined by the VITEK® 2 system (bioMérieux, France) (AST-P603) according to the CLSI guidelines (Clinical Laboratory Standards Institute, 2013).

The VITEK® 2 automated system, MALDI-TOF MS analysis and the M-PCR assay results were in agreement and all isolates were identified as *S. epidermidis*. All isolates (100%) were positive for the cefoxitin screen and showed resistance to benzylpenicillin and oxacillin. A total of 86% (*n* = 51/59) and 81% (*n* = 48/59) of isolates were resistant to erythromycin and gentamicin, respectively (Figure 1). All isolates (100%; *n* = 59/59) were susceptible to linezolid, teicoplanin, vancomycin and tigecycline (Figure 1).

The *ica*AB (*n* = 48/59) and *qac*A/B (*n* = 48/59) genes were both present in 81% of the isolates (Table 1). All isolates (*n* = 59/59) carried the *mec*A gene, whereas 83% (*n* = 49/59) of isolates carried the IS*256* element. Seventy-six percent (76%; *n* = 45/59) of the isolates harboured all four genes. The amplified *qac*A/B products (81%; *n* = 48/59) were digested with *Alu*I (New England Biolabs, USA). All isolates (100%; *n* = 48/48) harboured the *qac*A gene (220 bp product obtained after digestion).

**Table 1:** Molecular characterisation of *Staphylococcus epidermidis* isolates involved in CRBSIs.

**Patientnumber**	**Isolate number**	**Type ofspecimen**	**IS*256***	***ica*AB**	***mec*A**	***qac*A/B**	***mec*Acontrol**	***ccr*group**	***mec*group**	**Combination of the *ccr* group and the *mec* group**	**SCC*mec*type**	**MLST**
7	7.2	CVP	+	–	+	+	+	ND	ND	ND		
	7.3		+	+	+	+	+	4, 5	C	4,5C		
	7.4	BC	+	+	+	+	+	3,5	A	3,5A		
	7.5	BC	+	+	+	+	+	2	B	2B	IV	
	7.6	BC	+	–	+	+	+	2,5	B	2,5B		
9	9.1	CVP	+	+	+	+	+	5	A	5A		
	9.2	BC	+	+	+	+	+	2	B	2B	IV	
12	12.1	CVP	+	+	+	+	+	1	A	1A		
	12.3	BC	+	+	+	+	+	1	A	1A		
	12.4	BC	+	+	+	+	+	1	A	1A		
	12.5	BC	+	+	+	+	+	1	A	1A		ST54
14	14.1	CVP	+	+	+	+	+	4	A	4A		
	14.2	AC	+	+	+	+	+	3	A	3A	III	
	14.3	BC	+	+	+	+	+	3	A	3A	III	ST2
	14.4	BC	+	+	+	+	+	3,4	A	ND		
	14.5	BC	+	+	+	+	+	ND	A	*mec* class A		
18	18.1	BC	–	+	+	–	+	4,5	A	4,5A		
	18.2	BC	–	+	+	–	+	ND	A	*mec* class A		ST490
	18.4	CVP	–	–	+	–	+	2	B	2B	IV	
20	20.1	CVP	+	+	+	+	+	ND	A	*mec* class A		
	20.2	BC	+	+	+	+	+	1,2,5	A	1,2,5A		
22	22.1	CVP	+	+	+	+	+	2	B	2B	IV	
	22.2	BC	+	+	+	+	+	2	B	2B	IV	
26	26.1	CVP	+	+	+	+	+	2,5	B,C	ND		
	26.2	BC	+	+	+	+	+	2	B	2B	IV	ST28
27	27.1	CVP	+	+	+	+	+	3	A	3A	III	
	27.2		+	+	+	+	+	2,5	C	2,5C		
	27.3	BC	+	+	+	+	+	3	A	3A	III	ST2
28	28.2	CVP	+	+	+	+	+	2,4	B	2,4B		
	28.5	BC	+	+	+	+	+	2	B	2B	IV	
30	30.1	CVP	+	+	+	+	+	ND	B	*mec* class B		
	30.3	BC	+	+	+	+	+	ND	B	*mec* class B		ST2
36	36.1	AC	+	+	+	+	+	2,4,5	B,C	ND		
	36.2	BC	+	+	+	+	+	4,5	C	4,5C		
	36.3	BC	+	+	+	+	+	2	B	2B	IV	
38	38.1	CVP	+	–	+	–	+	1,5	B	1,5B		
	38.2	BC	–	–	+	–	+	1,5	B	1,5B		ST596
	38.3	BC	+	–	+	–	+	1,5	B	1,5B		
40	40.1	CVP	+	+	+	+	+	4,5	C	4,5C		
	40.2	BC	+	+	+	+	+	2	B	2B	IV	
	40.3	BC	+	+	+	+	+	2	B	2B	IV	
42	42.1	CVP	+	+	+	+	+	2,5	B,C	ND		
	42.5	BC	–	–	+	+	+	2	B	2B	IV	
43	43.2	CVP	+	+	+	+	+	2	B	2B	IV	
	43.3	VC	+	+	+	+	+	3	A	3A	III	
	43.4	VC	+	+	+	+	+	2	B	2B	IV	
	43.5	BC	+	+	+	+	+	2	B	2B	IV	ST2
	43.6	BC	+	+	+	+	+	4,5	C	4,5C		
	43.7	BC	+	+	+	+	+	2	B	2B	IV	
44	44.1	CVP	+	+	+	+	+	1	A	1A		
	44.2	BC	+	+	+	+	+	1	A	1A		ST54
53	53.1	CVP	–	–	+	–	+	5	C	5C	V	
	53.2	BC	–	–	+	–	+	5	C	5C	V	ST59
	53.3	BC	–	–	+	–	+	5	C	5C	V	
59	59.1	VC	+	+	+	+	+	2	B	2B	IV	
	59.2	BC	+	+	+	+	+	2	B	2B	IV	
60	60.1	CVP	+	+	+	+	+	2	B	2B	IV	
	60.2	BC	–	+	+	–	+	2,5	B	2,5B		
	60.3	BC	–	–	+	–	+	5	C	5C	V	

*The VITEK® 2 automated system (bioMérieux, France), MALDI-TOF MS (matrix-assisted laser deabsorption time of flight mass spectrometry) and ID PCR all identified the collected isolates as Staphylococcus epidermidis; AC, arterial catheter; BC, blood culture; CVP, central venous catheter; MLST, multilocus sequence typing; M-PCR assay, multiplex polymerase chain reaction assay; ND, no data; SCCmec, Staphylococcal Chromosome Cassette mec; ST, sequence type; VC, VasCath line; +, detected by M-PCR assay; –, not detected by the M-PCR assay. The shaded areas under the SCCmec type column indicate that certain isolates (n = 32/59) had multiple or unusual ccr and mec gene complex combinations and in these cases the raw ccr and mec gene combinations, instead of the SCCmec type, were reported. A Roman numeral was therefore not assigned to novel ccr and mec gene complex combinations if it is discovered in other methicillin-resistant staphylococci*.

### Molecular typing of *Staphylococcus epidermidis* isolates

SCC *mec* type IV (2B) was prevalent (31%; *n* = 18/59), followed by SCC*mec* type 1A (10%; *n* = 6/59) and SCC*mec* type III (3A) (9%; *n* = 5/59) (Table 1). Four isolates (7%; *n* = 4/59) harboured either SCC*mec* type V (5C2) or SCC*mec* type VII (5C1). The M-PCR assay by Kondo et al. (2007) did not distinguish between these two *mec* class C allotypes (i.e. *mec* class C1 and *mec* class C2) and it is therefore reported as SCC*mec* type 5C. Other *ccr* and *mec* gene classes detected included type VIII (4A) (3%; *n* = 2/59) and 5A (2%; *n* = 1/59). Only the *mec* gene complex could be detected in five isolates [*mec* class A (*n* = 3/5) and *mec* class B (*n* = 2/5)]. Three isolates harboured two *mec* groups (B,C) and multiple *ccr* groups [2,4,5B,C (*n* = 1/3) and 2,5B,C (*n* = 2/3)]. The remaining isolates (25%; *n* = 15/59) harboured multiple *ccr* gene complexes associated with a single *mec* gene complex [i.e., 4,5C (*n* = 4/15); 1,5B (*n* = 3/15); 2,5B (*n* = 2/15); 2,4B (*n* = 1/15); 2,5C (*n* = 1/15); 3,4A (*n* = 1/15); 3,5A (*n* = 1/15); 4,5A (*n* = 1/15), and 1,2,5A (*n* = 1/15)]. A single isolate was untypeable, even though it harboured the *mec*A gene. Only 46% (*n* = 27/59) of the isolates were typeable. The remaining 54% (*n* = 32/59) of the isolates had *mec-ccr* combinations that did not agree to the current classification scheme according to Kondo et al. (2007) and Ruppé et al. (2009). More than one *ccr* complex were detected in 31% (*n* = 18/59) of isolates.

**Figure 2:**
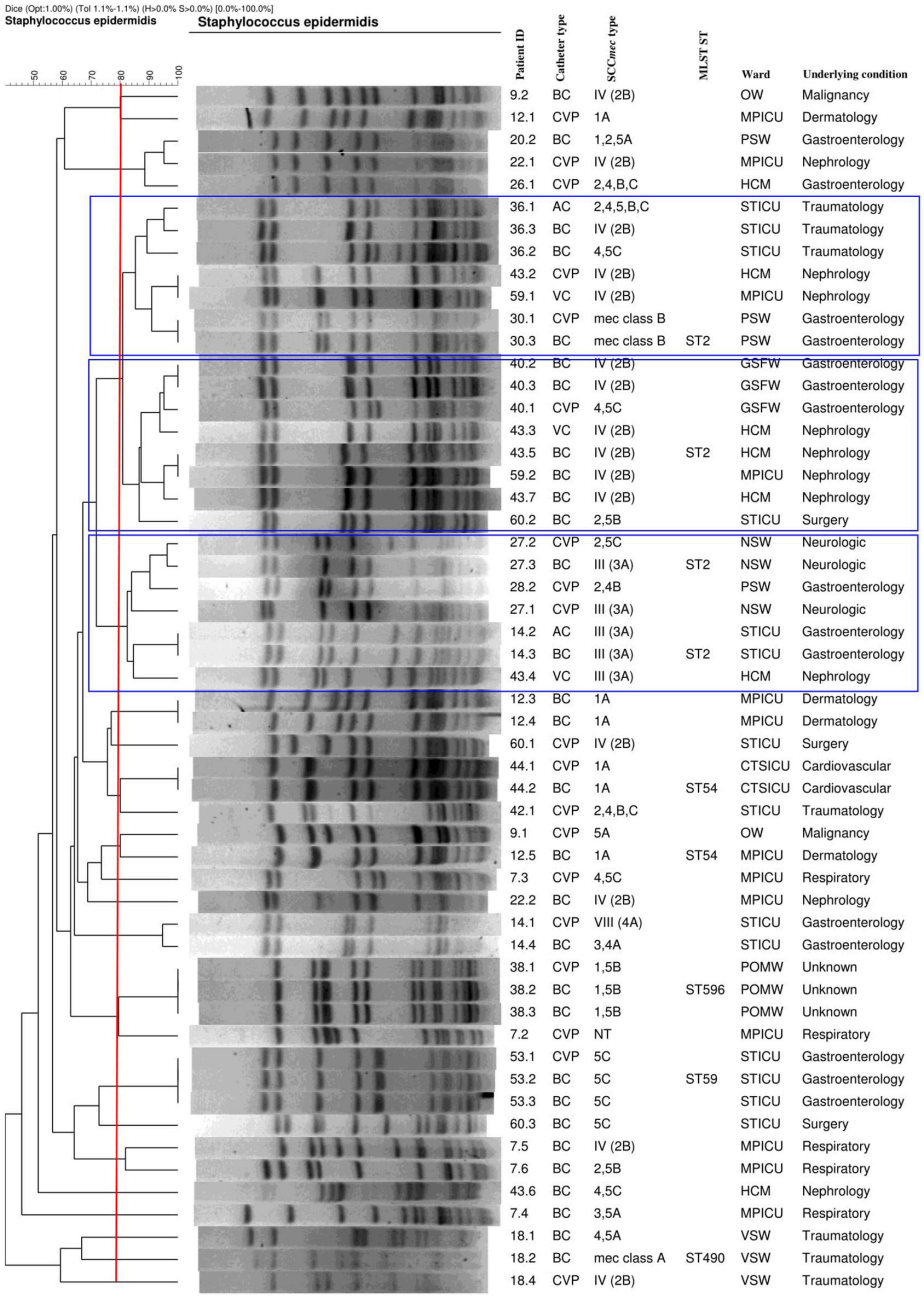
Genetic relatedness of catheter and blood culture *S. epidermidis* isolates implicated in CRBSI events (with the blue blocks representing the major pulsotypes). AC, arterial catheter; BC, blood culture; CTSICU, cardiothoracic surgery ICU; CVP, central venous catheter; GSFW, general surgery female ward; HCM, high care, multidisciplinary; MPICU, medical and pulmonology ICU; MLST, multilocus sequence typing; NSW, neurosurgery ward; NT, not typeable; OW, oncology ward; PSW, paediatric surgery ward; POMW, plastic/maxillofacial ward; ST, sequence type; STICU, surgery and trauma ICU; VC, VasCath; VSW, vascular surgery ward. Banding patterns of the catheter culture and BC isolates showing ≥80% similarity were clonal. Please note that isolates 14.5, 20.1, 26.2, 28.5, and 42.5 were untypeable and not included in the dendrogram.

**Figure 3:**
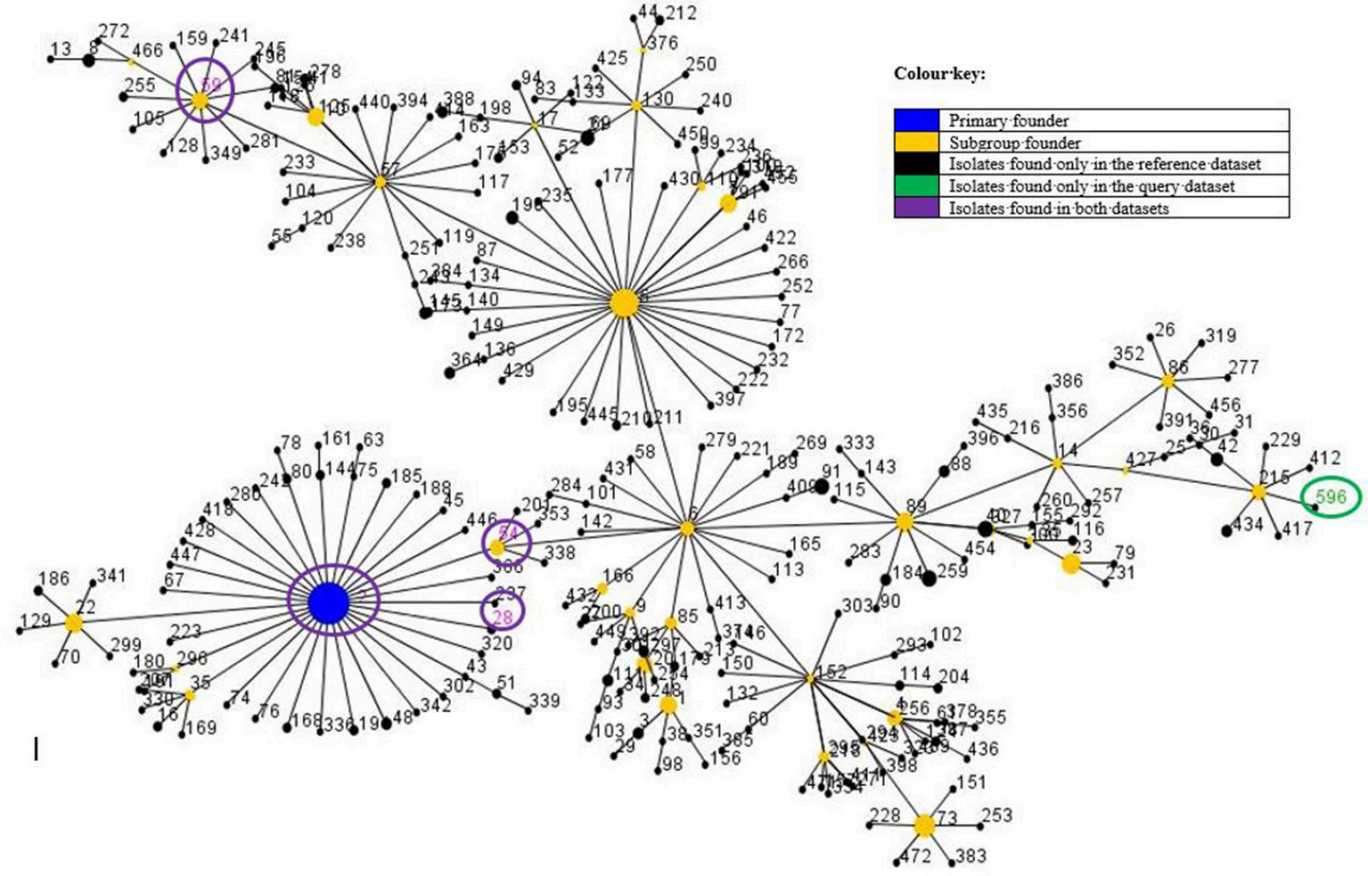
Comparative population snapshot of *S. epidermidis* STs detected in this study vs. STs in the *S. epidermidis* MLST database of clonal complex 2 as on the 16th of May 2017. Ten isolates were sequenced: ST2 (*n* = 4); ST54 (*n* = 2); ST28 (*n* = 1); ST59 (*n* = 1); ST490 (*n* = 1) and ST596 (*n* = 1). The sequence types (STs) found in this study are encircled, except for ST490 [not currently in the MLST database (last update 24-09-2015)]. The colour and meaning of each circle can be read according to the colour key.

The PFGE results showed three major pulsotypes, three minor pulsotypes and several singletons (Figure 2). The dominant ST among the 10 sequenced isolates was ST2 (40%, *n* = 4/10), followed by ST54 (20%; *n* = 2/10), ST28 (10%; *n* = 1/10), ST59 (10%; *n* = 1/10), and ST490 (10%, 1/10) (Table 1). A new ST was assigned to isolate 38.3 as ST596. A population snapshot of *S. epidermidis* is represented in Figure 3. The majority of isolates belonged to a single clonal complex [CC2], using eBURST analysis.

## Discussion

*S. epidermidis* is one of the most common isolated pathogens associated with CRBSI in this study. This is often due to the abundance of this opportunistic pathogen on the human skin, providing it with the ideal opportunity to contaminate IVC upon insertion. The molecular epidemiology and prevalence of the *ica*AB, IS*256, mec*A, and *qac*A/B genes in the *S. epidermidis* isolates (implicated in the CRBSI events) were described at a clinical setting in this study.

The level of resistance in *S. epidermidis* CRBSI isolates toward commonly prescribed antimicrobials, such as β-lactams, aminoglycosides, and macrolides, was high indicating multidrug resistance (MDR) as shown in Figure 1. The IS*256, ica*AB, and *qac*A/B genes were also highly prevalent among these isolates. All *S. epidermidis* isolates carried the *mec*A gene, which is similar to a study conducted in China (~96%) (Li et al., 2009), but higher compared to a study done in Portugal (~70%) (Rolo et al., 2012) and India (~67%) (Jena et al., 2017). The high degree of antimicrobial resistance and the presence of the *mec*A gene may indicate the presence of methicillin-resistant *S. epidermidis* (MRSE), which is more often resistant to other antimicrobials compared to methicillin-sensitive *S. epidermidis* (Otto, 2009). MRSE can also serve as a *mec*A gene donor to methicillin-sensitive *S. aureus*, which can lead to the emergence of methicillin-resistant *S. aureus* (MRSA) (Bloemendaal et al., 2010).

Schoenfelder et al. (2010) suggested that controlling MRSE may reduce the prevalence of MRSA. Infection prevention strategies, designed to decrease the incidence of CRBSI, include the use of antiseptics, such as chlorhexidine skin preparations prior to catheter insertion (Marschall et al., 2014). The *qac*A/B genes in *S. epidermidis* encode an efflux pump, which is associated with reduced susceptibility toward quaternary ammonium compounds and chlorhexidine (Horner et al., 2012; Wassenaar et al., 2015). According to the literature the *qac*A/B genes may indicate chlorhexidine failure but other unknown genes could be involved (Sekiguchi et al., 2004; Horner et al., 2012). The QacA and QacB efflux pumps have different substrate specificities (Wassenaar et al., 2015). The QacA efflux pump can transport a broad range of substrates, which include QACs, intercalating dyes (e.g., ethidium bromide) and cationic biocides (e.g., chlorhexidine), whereas the QacB efflux pump can transport only a limited range of substrates, which include QACs and intercalating dyes, but not cationic biocides (Wassenaar et al., 2015). Additional efflux pumps may also play a role (Otter et al., 2013).

The SCC*mec* types detected in the study were diverse, which are in agreement with other studies done in Finland and Western China (Ibrahem et al., 2008; Zong et al., 2011). The hospital environment is known to play a fundamental role in the amplification and the diversification of SCC*mec* elements in *S. epidermidis* (Rolo et al., 2012). *S. epidermidis* harbouring multiple *ccr* and *mec* gene complexes have been extensively reported in the literature (Ibrahem et al., 2008; Garza-González et al., 2010; Svensson et al., 2011; Zong et al., 2011; Rolo et al., 2012; Jena et al., 2017), but none of these studies have addressed the possibility of polyclonality. Polyclonality among *S. epidermidis* isolates has been reported in prosthetic joint infections (Galdbart et al., 1999), prosthetic valve endocarditis (Van Wijngaerden et al., 1997; Jena et al., 2017), healthy ocular surfaces (Ueta et al., 2007), and CRBSI (Rijnders et al., 2001). The overlapping of SCC*mec* types in isolates obtained from different specimens (patient 14 and patient 36) suggests the presence of a polyclonal *S. epidermidis* infection, due to the fusion of elements or due to the separate integration of various structural components (Ito et al., 2009; Shore and Coleman, 2013).

The PFGE results showed that the two STs (ST2 and ST54) were detected from different patients residing in different wards. These results were in agreement with a study done in Belgium at a clinical setting, by Cherifi et al. (2013) who also reported ST2 and ST54 as the most frequently detected clones. Du et al. (2013) conducted a study in China among patients, healthy volunteers and healthcare workers, who reported that the ST2 *S. epidermidis* clone was the most frequently detected among participants, while ST54 was only detected among healthcare workers. To the best of the authors knowledge, no literature could be found regarding *S. epidermidis* ST54 in Africa or South Africa. *S. epidermidis* isolates, obtained from different specimens (i.e., IVC and BCs), but from the same patient, were mostly clonal. However, some of the isolates collected from the same patient (patients 7, 9, 12, 20, 22, 26, 28, 43, and 60) were diverse (Figure 2). This could be explained by the introduction of multiple organisms residing on the skin as several patients had multiple catheters inserted or due to poor infection control measures at this setting (Table 1). The majority of ST2 strains harboured SCC*mec* IV with a single *ccr* group (2). This was similar to the results of Cherifi et al. (2013) who also detected SCC*mec* IV as the most dominant SCC*mec* type; however, multiple *ccr* complexes were reported by the authors. A new ST (596) was detected and assigned to one isolate [ID = 1031; isolate = UP2; country = South Africa] (https://pubmlst.org/bigsdb?db=pubmlst_sepidermidis_isolates&page=profiles).

Some of the study limitations included, that the results cannot be extrapolated to the rest of South Africa, since the study was done in a single centre, compliance toward the IDSA guidelines, in regards to BC submissions, is lacking, catheter cultures were not always accompanied by one or more BCs, which could have led to the inclusion of possible *S. epidermidis* contaminants. Due to limited funding all 59 *S. epidermidis* isolates could not be subjected to MLST.

## Conclusion

The isolated *S. epidermidis* isolates were MDR and all isolates carried the *mecA* gene. The high prevalence of the *mec*A gene and other virulence factors is worrisome since *S. epidermidis* can serve as a genetic reservoir to the more pathogenic *S. aureus*. The predominant ST in the studied hospital was ST2, followed by ST54 but a new ST (596) was also discovered. It is recommended that infection control and prevention strategies are intensified to limit the spread of *S. epidermidis* in the hospital environment. Further investigation is needed to determine resistance determinants toward chlorhexidine.

## Ethics statement

The study was approved by the Research Ethics Committee, Faculty of Health Sciences, University of Pretoria (Protocol number: 118/2013) and informed individual patient consent was waivered, since the study was observational and patient care was not influenced at any stage.

## Author contributions

ME, MK, and WS: conceived and designed the study; WS: collected all the clinical isolates; WS and ML: performed all the laboratory analyses; ME, ML, and WS: wrote the manuscript with critical appraisal and contributions received from all of the authors. All authors read and approved the final version of the manuscript.

### Conflict of interest statement

The authors declare that the research was conducted in the absence of any commercial or financial relationships that could be construed as a potential conflict of interest.
